# Th9 cells provide protective TB immunity

**DOI:** 10.3389/fimmu.2025.1581286

**Published:** 2025-10-06

**Authors:** Mei Xia, Azra Blazevic, Huan Ning, Christopher S. Eickhoff, Chad E. Storer, Richard D. Head, Jianguo Liu, Jessica Jarvela, David Stoeckel, Erin Rakey, Jan Tennant, Daniel L. Miller, Kathleen R. Holloway, Richard F. Silver, Daniel F. Hoft

**Affiliations:** ^1^ Division of Infectious Diseases, Allergy and Immunology, Department of Internal Medicine, Saint Louis University, St. Louis, MO, United States; ^2^ Genome Technology Access Center, Washington University School of Medicine, St. Louis, MO, United States; ^3^ Division of Pulmonary, Critical Care and Sleep Medicine, the Louis Stokes Cleveland VA Medical Center and Case Western Reserve University, Cleveland, OH, United States; ^4^ Division of Pulmonary, Critical Care and Sleep Medicine, Department of Internal Medicine, Saint Louis University, St. Louis, MO, United States; ^5^ Department of Pathology, Saint Louis University, St. Louis, MO, United States

**Keywords:** Th9 cells, IL-9, *mycobacterium tuberculosis*, BCG, bronchoalveolar lavage, transcriptomic assay

## Abstract

**Introduction:**

CD4+ Th9 cells have been associated with inflammatory and allergic diseases. IL-9/Th9 can function as both positive and negative immune regulators, but their protective effects against *Mycobacterium tuberculosis*(Mtb) are unknown. We found that Th9 cells were associated with mycobacteria-specific T cell responses primed by latent tuberculosis infection and BCG vaccination.

**Methods:**

To study TB-specific Th9 protective effects, we generated Th9 cells from ESAT6-specific TCR transgenic mice and healthy human donors.

**Results:**

Both murine and human Th9 cells significantly inhibited intracellular mycobacterial growth. In both *in vitro* models, IL-9 neutralization strongly reduced Th9 protective effects, and IL-9 treatment alone inhibited intracellular mycobacteria. ESAT-6-specific Th9 and Th1 cells were adoptively transferred into naïve Rag1/2^-/-^ recipients before aerosol Mtb infection. Th9 cells provided robust immunity as protective as Th1 cells, significantly reducing bacteria and pathologic changes post-infection. Differential persistence of Th9 vs. Th1 cell phenotypes was confirmed *in vivo*, and lung tissue qRT-PCR studies demonstrated the absence of IFN-γ responses in Th9-transferred mice, combined with unique expression of the Th9 specific markers IL-9, IL-10 and PU.1.

**Discussion:**

Th9 cells can provide important protection against Mtb infection, and should be targeted with future TB vaccine strategies. Furthermore, Th9 cells appear to utilize a novel protective mechanism independent from Th1-mediated protective responses.

## 1 Introduction

Tuberculosis (TB) remains a leading cause of global human morbidity and mortality due to a single infectious disease. According to the World Health Organization, 10.8 million people developed tuberculosis (TB) worldwide in 2023, almost half of whom never received treatment, and 1.25 million people died (1). The situation is worse for patients with multidrug-resistant TB and extensively drug-resistant TB (2). The concurrent epidemic of HIV in many countries contributes to the burden of TB deaths (3). The only available vaccine, *M. bovis* Bacillus Calmette-Guérin (BCG), is administered at birth and strongly protects against disseminated TB in children but confers more limited protection against pulmonary disease in (1, 4) adults. More effective vaccines are urgently needed; however, the development of new vaccines remains challenging. Tuberculosis (TB) vaccine development has focused largely on targeting T helper type 1 (Th1) CD4+ T cells. Disappointingly, despite inducing Th1 cells, the recombinant TB vaccine MVA85A failed to enhance protection against TB disease in humans (5). In addition, several animal studies (6–9) suggest that IL-17 producing T cells are efficiently generated following vaccination and are involved in the protective memory response against subsequent Mtb challenge. Human studies are less supportive of an important TB protective role for IL-17/Th17 responses. One report failed to show IL-17 being correlated with protection (10), while other reports suggest higher IL-17 responses are associated with TB disease (11, 12). In addition, recent research has expanded the paradigm of T-helper cell involvement in TB to include subsets with emerging roles, such as Th22 (13). These studies highlight the importance of exploring new and more effective pathways to improve vaccine-induced immunity against TB. Transcriptomic profiling in vaccine research serves as an excellent tool to find potential markers of vaccine‐induced responses that subsequently need confirmation at the protein or cellular level (14). Whole blood transcriptomic signatures that provide a broad view of the host response to TB have been able to differentiate patients with active TB from healthy controls, TB from other lung diseases, and even active TB from latent TB infection (LTBI) (12, 15–23). Bronchoalveolar lavage (BAL) cell transcriptomic signatures can provide profiles of local immunity to Mtb in LTBI subjects, in whom initial exposure to the organism via inhalation may be expected to result in optimal localization of responsive CD4+ T cells to the lung (24).

To better understand the coordinated systemic and mucosal immune responses involved in protection against TB, we performed RNAseq studies with samples of peripheral blood and BAL cells from healthy volunteers vaccinated with BCG and from people with latent TB infection (LTBI). IL-9 mRNA in activated blood CD4+ T cells topped the list of genes increased after intradermal BCG vaccination and in subjects with LTBI. Similarly, significantly increased Mtb-specific IL-9 responses were detected in BAL cells from subjects with LTBI or persons vaccinated with oral BCG, indicating induction of Mtb-specific Th9 memory responses with lung homing potential (25).

In recent years, T helper type 9 (Th9) cells have emerged as an independent Th cell subset that produce mainly IL-9, but also IL-10 and IL-21 (26, 27). They differentiate from naive CD4+ T cells in the presence of TGF-β1 and IL-4 (28, 29). Th9 cells play an important role in antitumor and helminthic parasite immunity and are associated with inflammatory responses (30–33). A few reports have shown an increase in IL-9 in pulmonary TB patients (34, 35). In addition, recently Kurtz et al. screened a panel of chemokines and lung cytokines collected at 14 weeks after BCG vaccination/Mtb challenge and have found that IL-9 is one of the strongest correlates of protection in the lungs of mice infected with Mtb (36). The role and functional relevance of Th9 cells in Mtb infection, however, remain largely unknown and require further examination.

In this study, we have investigated whether Th9 cells could protect against Mtb infection by generating Th9 cells differentiated from naïve ESAT6-specific TCR transgenic (Tg) CD4+ T cells and polyclonal blood CD4+ T cells from BCG-vaccinated human subjects. Using both models, our studies demonstrate that Th9 cells can significantly inhibit intracellular mycobacterial growth *in vitro*. Neutralization of IL-9 strongly reduced Th9 protective effects, and IL-9 treatment alone could inhibit intracellular mycobacteria. Adoptive transfer of Mtb-specific Th9 cells into naive Rag1/2^-/-^ hosts protected the recipient mice against Mtb challenge, providing immunity similar to Th1 cell adoptive transfer without apparent induction of IFN-γ responses detectable in lung tissues by qRT-PCR. Therefore, Th9 cells may provide unique protective mechanisms important for control of Mtb infection.

## 2 Results

### 2.1 Mtb-specific IL-9 responses are induced in BCG-vaccinated and LTBI subjects

**Figure 1:**
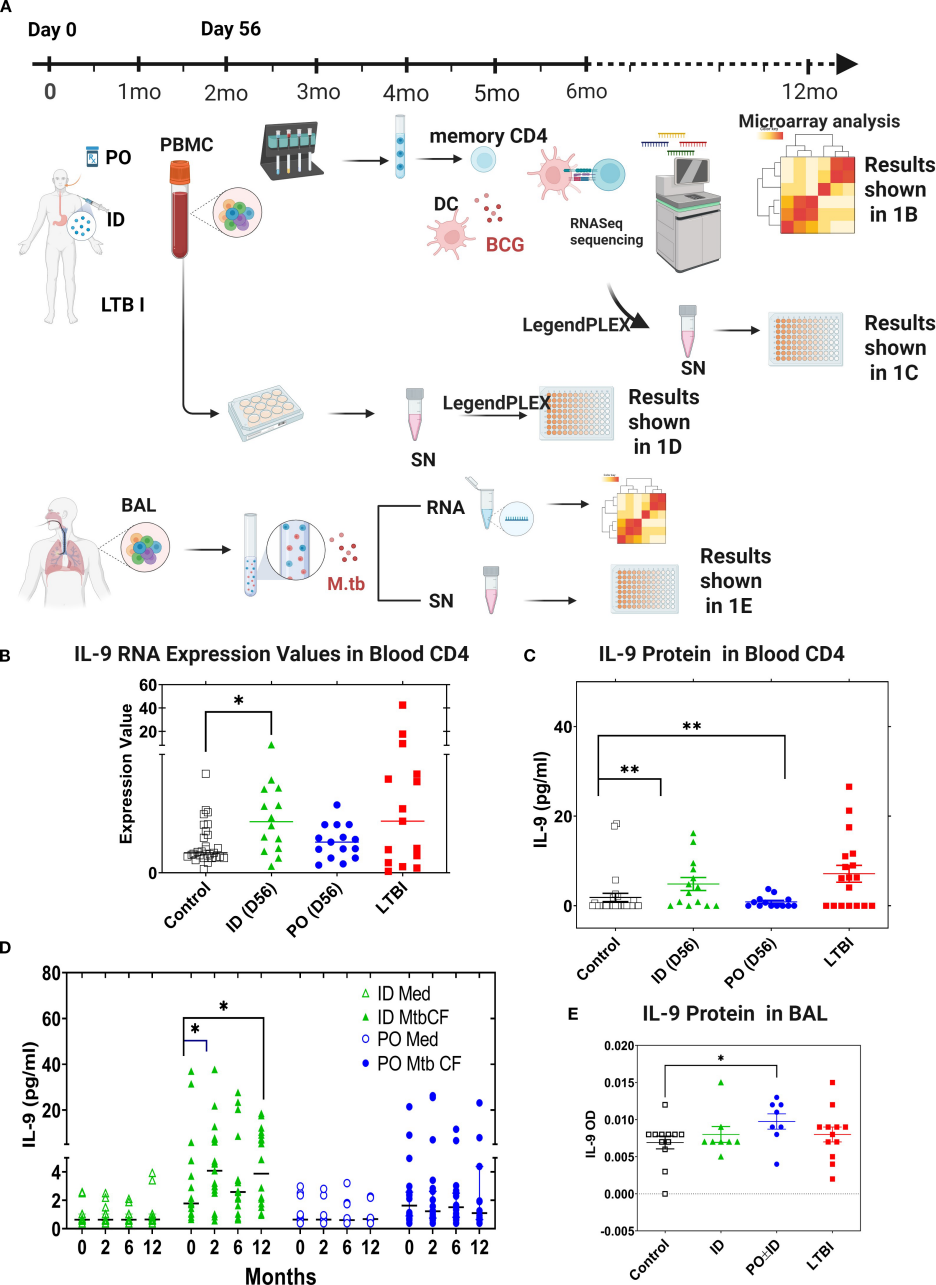
IL-9 expression is associated with BCG vaccine- and Mtb infection-induced TB immunity. **(A)** Schematic diagram to show the study groups, design. In studies of both peripheral blood CD4+ T cell responses and of cells obtained by BAL, gene expression of LTBI individuals and of recipients of ID or PO BCG vaccination were compared to that of Mtb/BCG-naïve subjects. In peripheral blood studies, pre-vaccination samples from subjects who later received ID or PO vaccination were pooled to form the naïve comparator group. Subsequent PBMC samples for all vaccinated subjects were obtained 56 days following their last vaccine dose. Memory CD4+ T cells were selected from thawed samples as were blood monocytes that were cultured *in vitro* with IL-4 and GM-CSF to develop autologous CD14^-^/CD11c^+^ monocyte-derived dendritic cells (MDDC). MDDC were then co-cultured with memory CD4+ T cells in the presence of BCG or in medium alone, and samples frozen for subsequent RNA extraction. In contrast, for the BAL cell sub-study (1A, lower panel), no subjects were recruited for bronchoscopy participation prior to being diagnosed with LTBI or receiving protocol BCG vaccinations; therefore, a separate group of Mtb/BCG-naïve individuals was recruited for bronchoscopy as controls. Unsorted BAL cells were then incubated overnight with virulent Mtb strain H37Rv or medium alone and frozen for eventual RNA extraction. For both sub-studies, RNASeq was performed, and data evaluated for comparisons between the study groups regarding both baseline gene expression and differential responses to *in vitro* infection. The different study groups are shown in black (controls), green (ID BCG), blue (PO BCG) and red (LTBI) throughout **(B–E)** Expression of IL-9 mRNA and production of secreted IL-9 protein from these same cultures are shown in **(B, C)**, respectively. **(D)** presents human IL-9 ELISA data produced in whole blood cultures stimulated with Mtb culture filtrate or incubated in medium alone, at different time points post vaccination. In **(E)**, BAL cells were infected with Mtb overnight and secreted IL-9 measured by ELISA. Median values for the different groups are compared. *p<0.05, **p<0.01 by Wilcoxon-matched pairs testing comparing post-vaccination to baseline responses.

We previously found that ID and PO BCG vaccination in humans induce differential mucosal and systemic trafficking patterns associated with distinct blood molecular signatures, but our sample sizes were too small for identification of the specific pathways/networks induced (37). Using the same *in vitro* co-culture model, we recently completed a systematic comparison of BCG and Mtb-induced gene expression, in both peripheral blood CD4+ T cells and unsorted BAL cells from recipients of PO vs ID BCG (25). Using the study scheme detailed in **Figure 1A**, we compared pre- and post-vaccination TB-specific blood CD4+ T cell responses in ID (n=20) and PO (n=20) BCG vaccine recipients, as well as in subjects with LTBI (n=20) as a positive control model of *in vivo* partial TB immunity. In addition to demonstrating upregulation of many genes previously recognized as contributing to Mtb immunity, we also made the unexpected observation that one of the most strongly upregulated genes in this assessment was IL-9 (25). IL-9 transcripts were significantly upregulated in peripheral blood CD4+ T cells from subjects previously vaccinated with ID BCG and subjects with a history of LTBI (**Figure 1B**). Additionally, significant IL-9 protein production was observed in blood T cells from ID BCG recipients and individuals with LTBI (**Figure 1C**). To further investigate IL-9 responses following BCG vaccination, we studied the levels of IL-9 protein present within the supernatants stored from heparinized whole-blood samples previously stimulated with mycobacterial antigens pre-vaccination and at 2-, 6- and 12-month post-vaccination time points (**Figure 1D**). These samples had been diluted 5-fold with medium and stimulated with Mtb culture filtrate (CF, complex lysate of all Mtb antigens secreted by exponentially growing organisms) for 4 days. Culture supernatants were subsequently analyzed by LEGENDplex and presented in **Figure 1D** to illustrate the time course of TB-specific IL-9 protein production during the first-year post-vaccination for subjects given ID BCG alone or PO BCG alone, presented as median values for 19 ID BCG recipients and 20 PO BCG recipients. Significant induction of IL-9 protein was observed in recipients of ID BCG at 2 months following vaccination in response to CF, and at both 6- and 12-months following vaccination in cultures stimulated with BCG. In contrast, no significant production of IL-9 was observed in whole blood from recipients of PO BCG at any time point in response to either stimulus. We also analyzed the IL-9 gene expression and protein production in BAL cell supernatants from vaccinated subjects, LTBI individuals, and BCG/MTN naïve controls. We observed that increased Mtb-induced IL-9 protein was produced only by BAL cells from recipients of PO BCG (**Figure 1E**). Overall, these results indicate that TB-specific IL-9 responses were present after BCG vaccination and in LTBI subjects. However, systemic and mucosal BCG vaccinations appeared to induce differential trafficking of Th9 cells to blood and lung immune compartments, respectively.

### 2.2 *In vitro* inhibition of mycobacterial growth by human and ESAT6-specific murine Th9 cells

**Figure 2:**
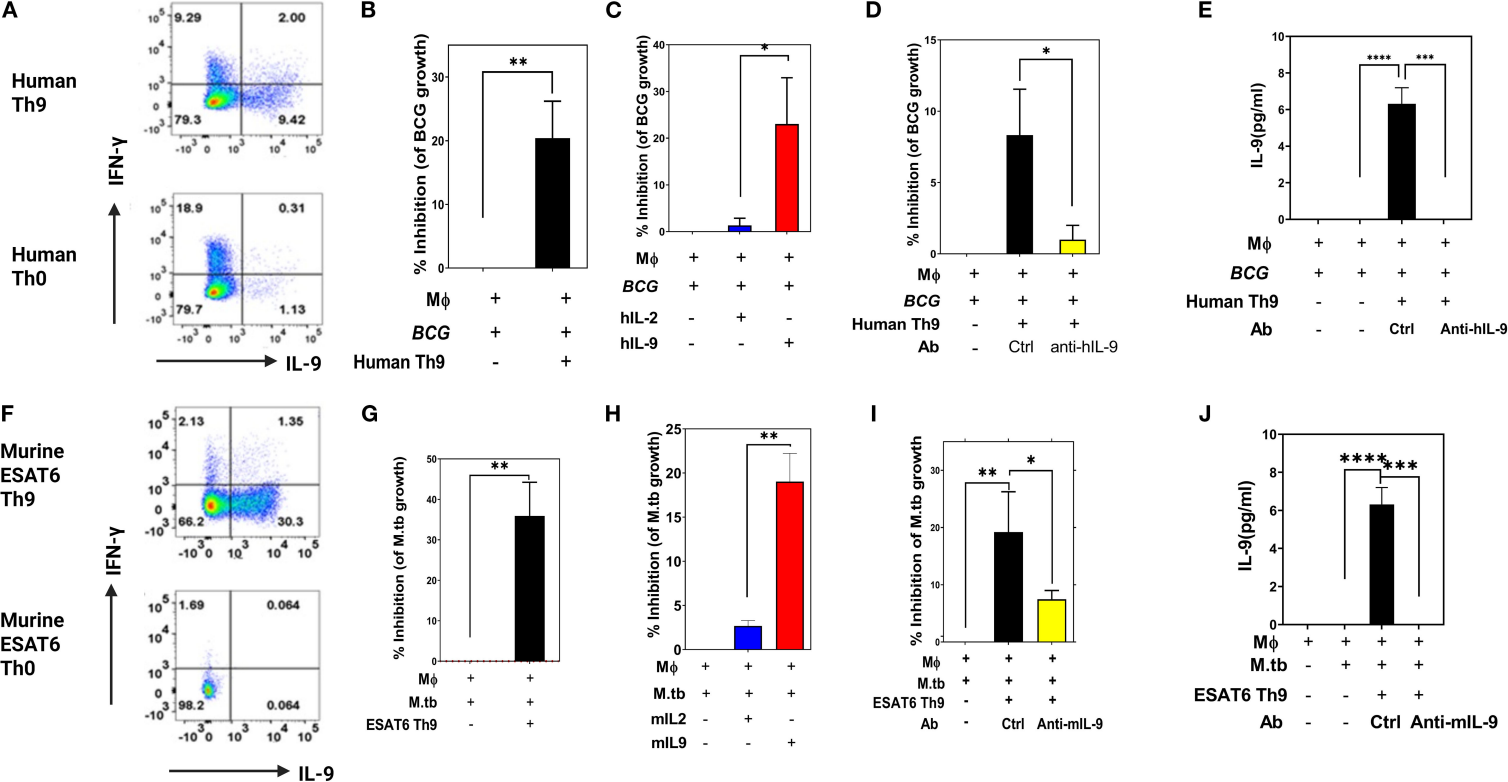
Protective *in vitro* effects of human and murine Th9 cells. Memory CD4+ T cells from PPD+ volunteers were activated with anti-CD3 and anti-CD28 in the presence of Th9 polarizing cytokines. Cells were collected on day 3 and intracellular production of IFN-γ and IL-9 was measured by ICS flow cytometry. Shown in **(A, F)** are representative human **(A)** and murine **(F)** experimental results, respectively. **(B–E)** show the protective *in vitro* effects of human Th9 cells and human recombinant IL-9. Blood monocyte targets were infected with BCG (MOI 3) overnight. Freshly differentiated human Th9 cells, purified human IL-9 and/or anti-IL-9 were added, and 3 days later BCG viability was determined by [^3^H] uridine incorporation. **(B–E)** show hTh9 effects, hIL-9 effects, the effects of IL-9 blockade, and IL-9 production by T cells in response to co-culture with infected monocytes, respectively. Both human Th9 cells and IL-9 alone significantly inhibited intracellular mycobacterial growth. **(G–J)** show the protective *in vitro* effects of murine Th9, murine IL-9 alone, blockade of IL-9 and production of IL-9 by stimulated T cells, respectively. Naïve CD4 T cells were purified from murine ESAT6-specific TCR Tg mice. Purified naïve CD4+ T cells were differentiated with anti-CD3 and anti-CD28 in the presence of Th9 polarizing cytokines. BMDM cultured from ESAT-6 TCR Tg mice were infected with Mtb at an MOI of 1 overnight. Both murine Th9 cells and IL-9 alone significantly inhibited intracellular Mtb replication *, represents p < 0.05, **, represents p < 0.01, ***, represents p < 0.001, ****, represents p < 0.0001 by Mann-Whitney U tests.

We next determined whether IL-9 and/or Th9 cells could contribute to protection against intracellular replication of mycobacteria in monocytes *in vitro*. Th9 cells were generated *in vitro* from BCG-vaccinated subjects and ESAT-6-specific TCR Tg mice. Memory CD4^+^ T cells were purified from PPD+/QuantiFERON- subjects and naïve CD4^+^ T cells were purified from ESAT-6-specific TCR Tg mice, and both were stimulated in plates coated with anti-CD3 anti-CD28 (BD). Th0 cells and Th9 cells were generated by addition of IL-2 alone or a combination of TGFβ1, IL-4 and anti-IFN-γ, respectively. On day 3 of culture, human and murine Th9 cell differentiations were confirmed via intracellular expression of IL-9 (**Figures 2A, F**, respectively).

As shown in **Figures 2B, C**, human Th9 cells as well as exogenously added IL-9 alone could inhibit the growth of BCG within human monocytes. Consistent with these results, **Figure 2D** demonstrates that the inhibitory effects of Th9 cells are abolished after neutralizing IL-9. We also evaluated IL-9 concentrations in these T cell and BCG-infected monocyte co-cultures (**Figure 2E**). As shown, significant IL-9 levels were observed in supernatants from Th9 cells co-cocultured with Mtb-infected monocytes. These data indicate that human Th9 cells exert IL-9-dependent protective effects inhibiting mycobacterial infection in monocytes.

We next examined whether *in vitro*-generated murine Th9 cells could inhibit or kill intracellular mycobacteria. To this end, ESAT-6-specific TCR Tg Th9 cells were generated and then co-cultured with Mtb-infected murine bone marrow-derived macrophages as target cells. As shown in **Figures 2G, H**, murine Th9 cells as well as exogenously added mIL-9 alone, significantly inhibited the growth of Mtb within murine bone marrow-derived macrophages. Notably, antibody-mediated neutralization of IL-9 in these cultures resulted in significant reduction of the ability of murine Th9 cells to inhibit intracellular mycobacteria (**Figure 2I**). As in our human studies, IL-9 was detected only in culture supernatants of Th9 cells stimulated with Mtb-infected monocytes (**Figure 2J**).

To rule out the possibility that IL-9 could have direct toxic effects on mycobacteria, we incubated various concentrations of BCG in Middlebrook 7H9 medium in the absence or presence with human or murine IL-9. These cultures were then assessed for both IL-9-induced short-term toxicity over 4 hours by the MTT assay, and for IL-9 effects on BCG growth in MGIT cultures comparing time-to-positivity (TTP). As demonstrated in **Supplementary Figure 1**, IL-9 by itself had no toxic effects on extracellular mycobacteria (**Supplementary Figure 1A**) and did not suppress BCG growth (**Supplementary Figure 1B**). Collectively, these results demonstrated that both human and murine Th9 cells have IL-9-dependent inhibitory effects on replication of mycobacteria in infected monocytes. In addition, as demonstrated in **Supplementary Figure 3**, we included a control Th0 cell group in our *in vitro* killing assay. The data clearly show that while Th0 cells provide a modest reduction in bacterial burden compared to the no-T-cell control, both Th1 and Th9 cells were significantly more protective. These data confirm that the enhanced protection is a function of the polarized effector phenotype, not merely the presence of antigen-specificity.

### 2.3 TB-specific Th9 cells protect mice against TB infection *in vivo*

**Figure 3:**
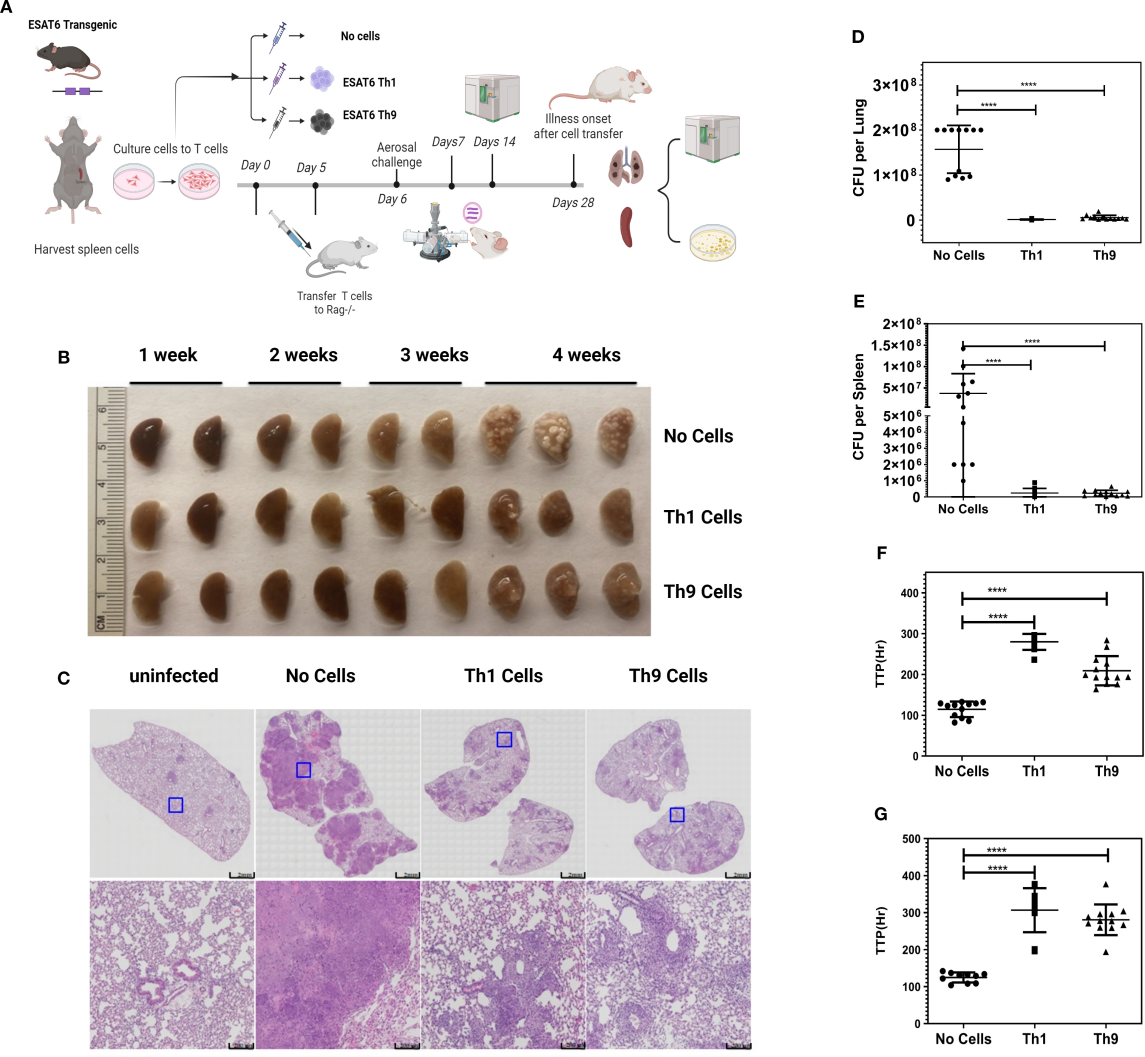
Adoptive transfers of Th9 cells provide *in vivo* protection against aerosol Mtb challenge. **(A)** presents a schematic diagram of the experiments. Naïve CD4+ T cells were isolated from the spleens of ESAT6-specific TCR Tg mice. Cells were differentiated into Th1 or Th9 cells *in vitro*. 5x10^6^ cells were injected i.v. into naïve Rag1/2^-/-^ recipients 1 d before Mtb infection. Groups of mice (N = 8–10 per group) were challenged with virulent Erdman Mtb, via aerosol exposure. Mice were sacrificed at various time points and lungs were harvested for the estimation of bacterial burden. **(B)** presents gross pathology in lungs of infected and treated mice. **(C)** shows histopathology in lungs from all mouse groups stained with H&E. Data shown are from one representative experiment. **(D, E)** show CFU and **(F, G)** show MGIT TTP studies of viable Mtb, from lungs **(D, F)** and spleens **(E, G)** from mice. Data shown are from one representative experiment (n = 5 mice/group). At least two independent experiments were performed for each endpoint. Error bars indicate S.D. p.i., post infection; *, represents p < 0.05, ****, represents p < 0.0001 by Mann-Whitney U tests.

We next performed adoptive transfer experiments to determine whether Th9 cells could protect mice against Mtb infection *in vivo*. To focus on the protective effects of Th9 cells, ESAT6-specific TCR Tg Th9 cells were adoptively transferred into Rag1/2^-/-^ mice, and then these mice were challenged with Mtb. To maximize Th9 cell differentiation *in vitro* and function after transfer, glucocorticoid-induced tumor necrosis factor receptor (TNFR)-related protein (GITR) was added during their *in vitro* differentiation. GITR is a member of the TNF receptor superfamily with co-stimulatory activity (38). Xiao et al. (39) verified that GITR-derived signaling favored the differentiation of Th9 cells. In addition, Kim et al. (40) found that GITR activation enhanced the antitumor effects of Th9 cells by reinforcing the function of DCs to elicit a stronger tumor-specific CTL response. Agonistic anti-GITR was used in our study to boost the Th9 cell differentiation and function *in vitro*. As shown in **Supplementary Figure 2**, stimulation by anti-GITR in combination with Th9 polarizing cytokines increased Th cell expression of IL-9 mRNA (**Supplementary Figure 2A**), and the frequency of Th9 cells (**Supplementary Figure 2B**). In addition, anti-GITR treated murine Th9 cells demonstrated enhanced inhibitory effects on the growth of Mtb within murine bone marrow-derived macrophages (**Supplementary Figure 2C**). **Figure 3A** includes a schematic diagram of these murine experiments. Rag1/2^-/-^ mice received adoptive transfer of Th9 cells, Th1 cells (positive control) or PBS alone (negative control), one day prior to challenge with Mtb via the aerosol route with a low-dose inoculum of ∼150CFU/mouse. The differentiation of ESAT-6-specific TCR Tg T cells into Th1 and Th9 cells was confirmed prior to transfer via intracellular expression of IL-9 and absence of prominent IFN-γ responses. Th9 cells generated with anti-GITR typically contained ~50% IL-9-expressing CD4+ T cells, with low expression of IFN-γ, IL-4, and IL-17 (data not shown). After resting Th1 and Th9 cells for an additional two days they were transferred i.v. into Rag1/2^-/-^ mice. The day after cell transfer, recipients were aerosol challenged with Mtb. Rag1/2^-/-^ mice not adoptively transferred with CD4+ T cells were infected with Mtb as controls. The kinetics of gross and histopathological alterations in the lungs were evaluated at weekly intervals throughout 28 days of infection (**Figures 3B, C**). Mice given Th1 or Th9 cells had marked reductions in numbers of granulomatous lesions and necrosis compared with control mice on day 28 (**Figure 3C**). In addition, general lung inflammatory reactions in mice given Th1 or Th9 cells were markedly improved compared with control mice (**Figure 3C**). Mice receiving Mtb-specific Th9 cells had significantly reduced bacterial burdens in their lungs and spleens as assessed both by CFU (**Figure 3D, E**) and MGIT time to positivity (TTP, **Figures 3F, G**). Collectively, these results indicate that similar to Th1 cells, Th9 cells can protect mice against TB infection *in vivo*.

### 2.4 Distinct inflammatory signatures are present in Th1 and Th9 adoptively transferred mice after Mtb infection

**Figure 4:**
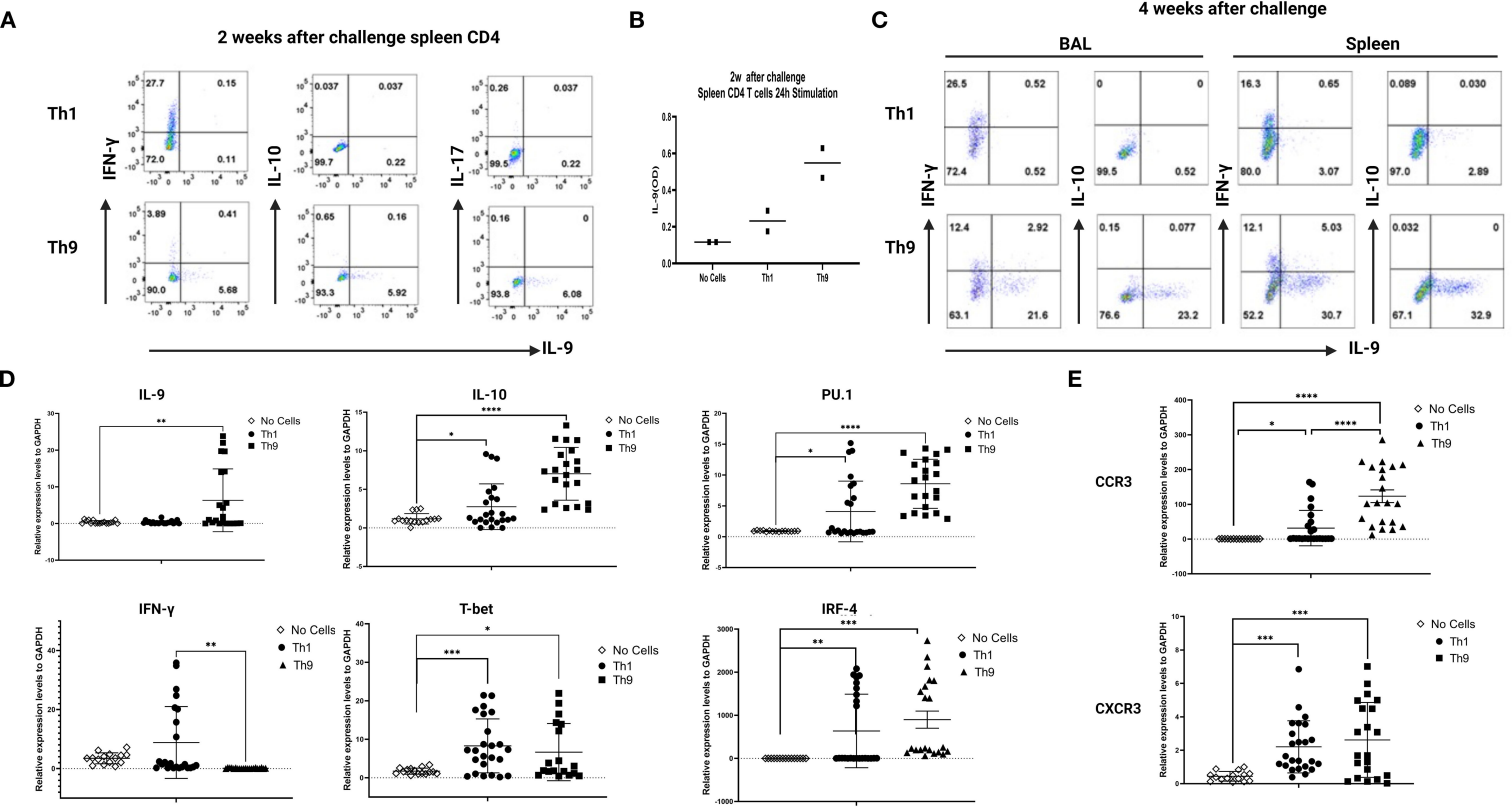
Distinct *in vivo* inflammatory signatures are associated with Th9- vs Th1- mediated protection. In **(A)**, purified spleen CD4+ T cells (50,000 per well) from mice 2 weeks after Mtb challenge were stimulated with Th9-polarizing conditions for 24 hours *in vitro*. Intracellular expression of IFN-γ and IL-9 was measured by ICS flow cytometry. **(B)**, culture supernatants collected from experiments described in A were analyzed for mouse IL-9 by ELISA. **(C)** presents BAL and spleen cells from mice harvested 4 weeks after Mtb challenge and studied as in A. Data are shown for one representative experiment. **(D)**, qRT-PCR analysis of IL-9, IL-10, PU.1, IFN-γ, T-bet and IRF-4. **(E)**, qRT-PCR analysis of CCR3 and CXCR3 in the 4wks post infection infected lungs from the different groups of mice. *, represents p < 0.05, **, represents p < 0.01, ***, represents p < 0.001, ****, represents p < 0.0001 by Mann-Whitney U tests.

To confirm differential persistence of Th1 vs Th9 cells *in vivo* after adoptive transfer and Mtb challenge, mice were sacrificed at 2- and 4-week post-infection, spleen cells were restimulated *in vitro* with ESAT6 overlapping peptides and both IFN-γ and IL-9 production were studied by flow cytometry. At both 2 and 4 weeks after infection the expected predominant Th1 vs Th9 phenotypes were confirmed after Th1 and Th9 adoptive transfer, respectively. At 2 weeks post challenge 10-fold higher percentages of CD4+ T cells making IFN-γ were seen in mice given Th1 cells and 10-fold higher percentages of CD4+ T cells making IL-9 were seen in mice given Th9 cells (**Figure 4A**). In addition, at 2 weeks IL-9 secreted protein levels were increased in Th9 adoptively transferred mice (**Figure 4B**). BAL cells are important for mucosal protection in the lungs, and responses by BAL cells serve as a potential surrogate marker of regional pulmonary TB immunity (Blazevic et al., 2014). At 4 weeks post-challenge similar percentages of BAL CD4+ cells from both groups produced IFN-γ, whereas Th9 transferred mice had 30-fold higher percentages of BAL CD4 T cells making IL-9 (**Figure 4C**).

Further analysis of the infected lung tissues from infected mice at 4 weeks post-infection was done by qRT-PCR with homogenized lung tissue. Results for mRNA expression of cytokines and relevant transcription factors revealed that only Th9 cell transfers were associated with significant induction of IL-9, IL-10 and PU.1/2, all of which have been previously reported as characteristic of a Th9 cell signature (**Figure 4D**). In contrast, in Th1 cell-transferred mice, mRNA expression of IFN-γ and T-bet were significantly increased in lung tissues compared with control mice (**Figure 4D**). Importantly, IFN-γ expression was not detected in the lungs of Th9 transferred mice, indicating that Th9-mediated protection was independent of a classic Th1 response (**Figure 4D**).

We next sought to identify how Th9 cells home from blood to Mtb-infected lung tissues. Therefore, in the current study, we examined chemokine receptor usage by Th9 cells and determined the homing receptors involved in their recruitment to sites of infected lungs. Results for mRNA expression of chemokines and their receptors revealed that Th9 cell treatment was associated with significant increases in the expression of both CCR3 and CXCR3 in lung tissues (**Figure 4E**) but not CCR6 (not shown). In the Th1 cell recipient mice, mRNA expression of receptors including CCR3 and CXCR3, were also increased in the lung tissues as compared with control mice, as reported previously. This data strongly indicate that Th9 cells induced protection involving a mechanism distinct from classic Th1 protective effects.

## 3 Discussion

A deeper understanding of the immune responses associated with protective TB immunity is required to enable the development of more effective vaccines and therapeutics. To date, IFN-γ-producing Th1 cells have been an important focus for studies of TB immunity, appropriately so because Th1 cells have been established as vital for optimal containment of Mtb and have been extensively studied for development of novel vaccines and therapeutics against Mtb infection (41–43). However, recent studies demonstrate that Mtb-specific Th1 cells alone do not predict protection in humans against TB (5). Therefore, although Th1 cells are required for control of Mtb replication, other types of immune cells will be needed to prevent infection and eradicate the bacteria.

In the past decade, we have witnessed an explosion of new approaches and technologies allowing the exploration of the human immune system (44). Proof-of-concept studies demonstrating the use of systems biology approaches to identify molecular signatures of protective vaccination responses used the yellow fever live-attenuated vaccine YF-17D as an initial model (45, 46). Subsequently, several groups applied systems-based analysis to study immune responses induced by vaccines for seasonal influenza (47, 48), meningococcal disease (49), shingles (50), malaria (51), smallpox (52), Ebola vaccines (53), and a candidate vaccine against HIV (54). We previously identified differential mucosal and systemic immune trafficking of vaccine responses induced by PO and ID BCG, respectively, and also reported distinct TB-specific blood gene expression signatures associated with this differential trafficking in recipients of PO and ID BCG (37). In human studies, sampling of airway immune cells via bronchoalveolar lavage provides a safe, well-tolerated means to assess local immunity within the lung. Cells of the airway also provide the first line of defense against infection with respiratory pathogens such as Mtb. More recently, we have compared systemic blood responses and mucosal lung responses in subjects with LTBI and recipients of PO vs ID BCG (25). A systematic comparison based on global BCG and Mtb-induced gene expression was performed not only in peripheral blood CD4+ T-cells but also on unsorted BAL cells. Although ID BCG and LTBI induced potent mycobacteria-specific T cell responses detectable in blood, only subjects with LTBI and recipients of PO BCG developed vaccine-induced TB-specific memory immune responses localized to the lung (25). In this work, we were surprised to find that IL-9 expression at both the transcriptional and protein levels were reproducibly associated with the strongest vaccine- and LTBI-induced responses in both mucosal and systemic immune compartments. Because of these findings we have conducted the work reported here to determine whether IL-9 and/or Th9 cells could contribute to protection against intracellular replication of mycobacteria in monocytes, and whether they can provide protection against Mtb infection *in vivo*.

Th9 cells are a newly described T-helper subset that have pleiotropic functions during inflammation and protective immunity. A study conducted by Ye et al. investigated the differentiation and recruitment of Th9 cells stimulated by pleural mesothelial cells during human Mtb infection. Their data indicated that increased Th9 cells with an effector memory phenotype were found in TB-associated pleural effusions (35). Another pilot study reported that the percentages of Th9 and Th22 cells were able to distinguish TB pleuritis from malignant pleural effusions (55). The role and functional relevance of Th9 cells in Mtb infection, however, have remained largely unknown. In our current study, Th9 cells generated from PPD+ volunteers, and IL-9 itself without any direct cytotoxic effect on BCG or Mtb infected monocytes *in vitro*, provided protection like the levels seen with Th1 cells. We also studied the capacity of Th9 cells differentiated from murine ESAT6-specific TCR Tg T cells to inhibit Mtb growth both *in vitro* and *in vivo*. Th9 cells were generated from human polyclonal CD4 T cells and naïve ESAT6-specific TCR Tg T cells using IL-4 and TGF-β. These Th9 cells significantly inhibited intracellular mycobacterial growth in human and syngeneic murine macrophages *in vitro*. Neutralization of IL-9 strongly reduced Th9 protective effects, and IL-9 alone could inhibit intracellular mycobacteria. In addition, ESAT-6-specific Th9 cells were transferred into naive recipients before aerosol Mtb challenge to evaluate the ability of Th9 cells to protect against TB *in vivo*. We report that Th9 cells can protect mice as well as Th1 cells, resulting in 100-fold fewer bacteria in infected lungs and spleens despite the absence of detectable IFN-γ responses in the challenged lung tissues.

To date, no rapid and robust method has been developed to isolate viable Th9 cells directly ex vivo, based on their ability to secrete IL-9. To focus on the role of Th9 cells in protection after adoptive transfer into Rag1/2^-/-^ mice, it was essential to maximize Th9 cell differentiation and function *in vitro*. Glucocorticoid-induced GITR, a co-stimulatory member of the TNF receptor superfamily, enhances immune responses by interacting with TNFR pathways (38). Xiao et al. (39) demonstrated that GITR signaling enhances Th9 cell differentiation via specific transcription factors including STAT6, BATF, PU.1, IRF-4, and the canonical NF-κB pathway involving p50-RelA.verified that GITR-derived signaling favored the differentiation of Th9 cells via induction of distinct transcription factors not including Foxp3 involved in Treg cell development. Specifically, GITR-induced IL-9-producing cells and required the involvement of STAT6, BATF, PU.1, and IRF-4, and activation of the canonical NF-κB pathway (p50-RelA). These factors bind to the Foxp3 promoter, recruiting histone deacetylases to reduce chromatin accessibility. This process can inhibit Foxp3 expression while simultaneously enhancing IL-9 expression. This mechanism enhances immune responses by promoting Th9 differentiation while inhibiting regulatory T-cell development. In addition, Kim et al. demonstrated that GITR activation improves Th9 cell antitumor effects by strengthening dendritic cell function, leading to a more robust tumor-specific CTL response (40). Anti-GITR was used in our study to boost the Th9 cell differentiation and function *in vitro*. ESAT6-specific Th9 cells were increased from 20% to 50% after anti-GITR treatment. Furthermore, the Th9 cells generated with addition of anti-GITR could significantly inhibit intracellular mycobacterial growth in syngeneic murine macrophages *in vitro*. Given its Th9-promoting effects, anti-GITR was systematically applied across all experimental conditions, including both *in vitro* and *in vivo* models.

The stability of the Th9 cell subset *in vivo* remains incompletely understood (56, 57). Current understanding of Th9 cell plasticity is derived from studies utilizing adoptive transfer of *in vitro* differentiated Th9 cells. Experimental evidence shows these cells can become pathogenic by converting into cells that produce IFN-γ and IL-17 rather than their original IL-9 secretion (58). This phenotypic instability was further demonstrated in ocular inflammation models using HEL-specific Th9 cells, where infiltrating lymphocytes predominantly produced IFN-γ rather than IL-9 at disease sites. Notably, IL-9 blockade failed to prevent pathology in this model, implicating cytokine switching rather than IL-9 itself in disease pathogenesis (59). Multiple investigations have documented Th9-to-Th1 conversion in various inflammatory contexts (28, 60, 61). However, other experimental systems report consistency of IL-9 production following transfer, with IL-9 neutralization abolishing Th9-mediated effects (62–64). In our Murine TB challenge model, adoptively transferred Th9 cells maintained their capacity to produce IL-9 and IL-10 production in spleens during the first month after TB infection without the development of IFN-γ- or IL-17-producing T cells. In contrast, mice receiving Th1 cells produced high levels of IFN-γ responses detectable in infected lung tissues not seen in Th9 recipients. Further analysis of infected lung tissues from Th9 recipients by qRT-PCR identified cytokines and transcription factors associated with Th9 cell but not Th1 cell responses including expression of IL-9, IL-10 and PU.1 while Th1 recipients showed expected IFN-γ dominance in pulmonary lesions.

CD4+ T cells play a crucial role in controlling *Mycobacterium tuberculosis* (Mtb) infection by engaging infected macrophages and limiting bacterial proliferation. For optimal antimicrobial activity, these T cells must efficiently migrate from lymphoid organs to infected lung tissues. This trafficking is guided by chemokine receptor programs established during T cell activation, which shape both functional specialization and tissue-specific homing (65). Different CD4+ subsets—including naïve, Th1, Th2, Th17, and regulatory T cells—exhibit distinct chemokine receptor expression patterns that dictate their migration to specific sites of inflammation (65, 66). As described previously (67), most Th9 cells co-express CCR3, CXCR3, and CCR6, enabling their recruitment to diverse inflammatory environments. Their trafficking behavior varies by context: in allergic inflammation, Th9 cells rely on CCR3 and CCR6 (but not CXCR3) to accumulate in the peritoneal cavity, whereas during neuroinflammation, they utilize CXCR3 and CCR6 (but not CCR3) to infiltrate the CNS. Also, it has been reported that in tuberculous pleurisy, pleural mesothelial cells promote Th9 cell recruitment through CCL20-CCR6 interactions (35). Therefore, in the current study, we examined chemokine receptor usage by Th9 cells and determined the homing receptors involved in their recruitment to sites of infected lungs. Results from mRNA expression of chemokines in the infected lungs revealed that Th9 cells were associated with increased expression of CCR3 and CXCR3 in infected lung tissues, but not CCR6, suggesting that CCR3 and CXCR3 are important in our model for trafficking of Th9 cells to the infected lungs. Therefore, our work indicates that in Mtb-infected mice, antigen-specific CD4+ Th9 cells expressing CXCR3 and CCR3 become localized to the lung parenchyma and provide efficient control of bacterial growth *in vivo*.

In addition, this study still has some limitations. Our data within the Rag^-^/^-^ model demonstrates that polarized Th9 cells are as protective as Th1 cells via a distinct, IFN-γ-independent mechanism, supporting a protective role for Th9 cells. Future studies will need to confirm that Th9 cells can protect in immunocompetent mice. The mechanism by which IL-9 regulates anti-TB functions was not identified. IL-9 is also known to enhance macrophage antimicrobial functions, including phagolysosomal fusion, ROS production, and the expression of antimicrobial peptides. Therefore, the net effect of IL-9 in TB may represent a critical balance between its capacity to regulate damaging inflammation and its potential to directly bolster antibacterial defenses, a balance that is likely tipped by the local cytokine milieu and disease stage. While the current study indicates the protective role and sufficiency of IL-9 for this protection, IL-9 KO mice are being used in our ongoing studies to future define this precise mechanistic pathway.

Taken altogether, these data advance our knowledge of a distinct role for antigen specific Th9 cells in TB immunity. This novel understanding advances the goal of developing improved TB vaccines for humans. More broadly, these studies provide the first clear evidence that prophylactic and immunotherapic vaccines that trigger Th9 cell development, and/or treatment with IL-9 alone, may provide important new strategies for enhanced control of TB and other global public health problems. Further investigations are ongoing to identify the more detailed mechanisms of protection mediated by Mtb-specific Th9 cell responses.

## 4 Materials and methods

### 4.1 Subject enrollment

The clinical trial (DMID-01-351) involved 68 subjects randomized into a double-blind, placebo-controlled comparison of Danish or Connaught BCG given intradermally (ID), orally (PO) or by both routes, was described previously (37). These subjects had all undergone vaccination within 5 years of participation in research bronchoscopy procedures. For the purposes of our Bronchoalveolar Lavage (BAL) studies, subjects were divided into two groups: those who had received ID BCG vaccination only, and those who had received oral BCG vaccination, with or without ID BCG. LTBI subjects were self-identified on the basis of prior positive PPD skin test or QuantiFERON TB Gold In-Tube (QFN-GIT) blood test as described previously (24). All human subjects’ protocols were reviewed by the IRBs of each participating institution.

### 4.2 BAL

Bronchoalveolar lavage (BAL) cells were obtained from four human subject groups (negative controls, latently Mtb infected, PO BCG vaccinated, and ID BCG vaccinated). Subjects from previous PO/ID BCG trials performed at SLU were recruited for a single bronchoscopy with BAL. Bronchoscopies of LTBI and control subjects were performed in the Dahms Clinical Research Unit (DCRU) at University Hospitals Cleveland Medical Center. At both sites, bronchoscopy and BAL were performed and samples processed using previously described protocols (68). Briefly, BAL samples were obtained by instillation and subsequent withdrawal of up to eight 30 mL aliquots of pre-warmed buffered saline. Recovered BAL fluid was placed on ice for transport to the laboratory. Samples were aliquoted into 50 ml polypropylene tubes and immediately centrifuged at 2000 RPM (480 x g) for 10 minutes. BAL cells were stained and counted using a hemocytometer. Following counting, BAL samples were resuspended in 4 ml of IMDM with 30% autologous serum (AS) and 1% penicillin G and aliquoted into sterile screw topped microfuge tubes (Sarstedt, #72.692.005, Newton, NC) with at least 1x10^6^ cells per tube.

### 4.3 Mice

ESAT-6-specific TCR Tg mice were originally generated as described above at the Van Andel Institute and subsequently back-crossed onto C57BL/6J (69). ESAT-6-specific TCR Tg mice were screened for expression of Vβ6 and ESAT-6 tetramer staining on CD4+ T cells from the peripheral blood. Mice were housed in pathogen-free conditions in the Department of Comparative Medicine at Saint Louis University. Experimental mice were age- and sex-matched and used between 8 and 12 weeks of age. Rag1/2^-/-^ mice were obtained from the Jackson Laboratory and housed in sterile pathogen-free conditions. Rag1/2^-/-^ mice used in adoptive transfer experiments were 4- to 6-weeks-old.

### 4.4 Cell preparation and infection for RNASeq

To perform the study of the molecular signatures of peripheral blood CD4+ T cells induced by ID vs PO BCG vaccination and LTBI, we purified CD4+ T cells with Miltenyi immunomagnetic beads and prepared dendritic cells (DC) for use as antigen presenting cells as described previously (37). Autologous DC were infected or not with Danish BCG (MOI of 20) and then co-cultured overnight with memory CD4+ T cells purified from pre- (D0) and post-vaccination (D56) PBMC. The supernatants from the co-cultures were collected for cytokine assays (below). Total BAL cells were infected 2h with Mtb H37Rv (No. NR-123; BEI Resources, Manassas, VA) at a multiplicity of infection (MOI) of 3 as previously described (24). Extracellular bacilli were washed away, and then infected cells were cultured at 37°C with 5% CO2 for an additional 24h. Supernatants were collected for later use in assessing Mtb-induced cytokine production (below). 5–10 volumes RNAlater (Qiagen Cat# 7020 Valencia, CA, and USA) were added to the pelleted cells according to manufacture recommendations prior to transfer to −80°C storage until use in RNA preparation.

### 4.5 RNA extraction and quantification

Total RNA was extracted from all samples (co-cultured memory CD4 T cells, infected BAL cells) for gene expression analysis. The RNA from memory CD4 T cell co-cultures was extracted with RNAeasy Mini Kit (Qiagen Cat# 74104, Valencia, CA, and USA). Therefore, pre-, and post-vaccination samples were studied for each BCG-vaccinated volunteer – and samples from a single time point were studied for each LTBI volunteer. Frozen cell pellets in RNA later were equilibrated to room temperature before starting the procedure, cells were washed by adding the same amount of PBS and centrifuged to obtain pellets. 700 µl QIAzol Lysis Reagent (Qiagen Cat# 79306, Valencia, CA, and USA) was added to the pelleted cells and RNA was extracted by using a miRNeasy Mini Kit (Qiagen Cat# 217004, Valencia, CA, and USA) as per the manufacturer’s instructions. RNA quality was assessed on an Agilent 2100 Bioanalyzer and samples with an RNA Integrity Number (RIN) < 6 were excluded from further analyses. The RNA concentration was determined using a Quant-iT™ RiboGreen^®^ RNA Assay Kit (Thermo Fisher Scientific). RNA samples with RIN Values greater than 8 were submitted to the high throughput Microarray Core at Washington University where libraries were prepared as follows.10 ng of total RNA was used as input for cDNA preparation with the SMART-Seq v3 Ultra Low Input RNA Kit for Sequencing (Clonetech) according to the manufacturer’s specifications. Multiplexed samples were sequenced twice according to the protocol for 150 base pair (bp) reads utilizing NovaSeq S4.

### 4.6 Human IL-9 ELISA

IL-9 concentrations were determined using human IL-9 ELISA kits (Biolegend). Culture supernatants from BCG-stimulated peripheral blood CD4+ T cell cultures and Mtb-infected BAL cells were separated and subjected to ELISA assays following the manufacturer’s instruction. Statistical analyses were performed using Mann-Whitney U tests for unmatched group comparisons.

### 4.7 *In vitro* T cell differentiation

Naïve CD4+ T cells were purified from the spleens of ESAT6-specific TCR Tg mice (Miltenyi Biotec Cat# 130-104-453) according to the manufacturer’s protocol. Purified ESAT6-specific TCR Tg naïve CD4 + T cells were cultured in plates coated with 1ug/ml anti-CD3 (Clone 145-2C11) and 0.5ug/ml anti-CD28 (Clone 37.51). To generate Th9 cells, cells were treated with 20 ng/ml IL-4 (BD), 2 ng/ml hTGF-β1 (Biolegend), and 10 μg/ml anti-IFN-γ (R46A2, Bio X cell). To generate Th1 cells, cells were cultured with 20 ng/ml mIL-12 (Biolegend), 10 U/ml mIL-2 (Roche), and 10 μg/ml anti-IL-4 (11B11, Bio X cell). Cells were grown at 37°C under 5% CO2 and then transferred to a new plate on day 3 of culture and 50 U/ml IL-2 was added (R&D Systems). On day 3 and day 5 of culture, Th9 cell differentiation was confirmed via intracellular expression of IL-9. Murine Th9 and Th1 cells were washed three times with PBS before all downstream assays. A similar protocol was used to make human polyclonal Th9 CD4 + T cells. Human PBMCs were prepared from heparinized venous blood by Ficoll- Hypaque density gradient centrifugation. Human memory CD4+ T cells were isolated from human PBMCs using magnetic separation (Miltenyi Biotec Cat# 130-091-893). These memory CD4 T cells were cultured in plates coated with 1ug/ml anti-CD3 (OKT3, Bio X cell) and 0.5ug/ml anti-CD28 (CD28.2, Bio X cell) to generate Th0 cells with only 10 U/ml hIL-2, and Th9 cells with 20 ng/ml hIL-4 (R&D Systems), 2 ng/ml hTGF-β1 (Biolegend), and 10 μg/ml anti-IFNγ (R&D Systems) and grown at 37°C under 5% CO2. After 3 days cells were transferred to new plates and 50 U/ml IL-2 was added (R&D Systems). On days 3 and 5 of culture, human Th9 cell differentiation was confirmed via detection of intracellular expression of IL-9 by flow cytometry.

### 4.8 Human monocyte-derived macrophages and mouse bone marrow-derived macrophages

Human peripheral blood mononuclear cells (PBMCs) were obtained from healthy Tuberculin skin test negative donors by leukapheresis. Monocytes were purified from PBMCs by plastic-adherence based on the unique adhesion properties of monocytes/macrophages among PBMC populations (70). Briefly, 1.5 x 10^5^ PBMCs were plated in each well of 96-well round-bottom microtiter plates (Corning Inc. Costar-3799). Non-adherent cells were washed off with complete RMPI-1640 medium after 2h or overnight incubation at 37°C and 5% CO2. In general, approximately 10% of the initial number of PBMCs plated were retained as adherent monocytes (>90% CD14+, data not shown), which were then cultured one more day before infection with BCG or Mtb. Bone marrow (BM) cells were isolated from femora of ESAT6-specific TCR Tg mice as previously described (70). To generate BM-derived macrophages (BMDM) cells (2.5 × 10^4^ cells/well) were cultured in 10% FBS DMEM medium (D10) in 96-well plates (Corning Inc. Costar-3595) for 7 days with 20ng/ml murine recombinant M-CSF (PeproTech 315-02). After 7 days of culture, BMDM formed a confluent monolayer, and the cell density was estimated to be 2.5 × 10^5^ cells per well in 96-well plates.

### 4.9 Assay of T cell-mediated inhibition of intracellular mycobacterial growth

The assay was performed as previously described (71) with minor modifications (67–72). Briefly, adherent human or murine monocytes were infected overnight with BCG or Mtb at MOI of 3 and then extracellular BCG/Mtb were washed away. Human or murine Th9 cells were added to achieve an effector to target ratio of 3:1. Co-cultures were incubated at 37°C with 5% CO_2_ for 72h. Supernatants were collected for the IL-9 ELISA. The monocytes were lysed with 0.2% saponin in RPMI 1640 medium and released viable BCG/Mtb were quantified by [^3^H]-uridine (Revvity Health Sciences, Inc) incorporation. The percentages of bacterial growth inhibition were determined using the formula: % inhibition = 100 - [100 ´ (DPM in the presence of T cells lines/DPM in the absence of T cells)]. In some assays, a lower density of adherent monocytes was used to assess the inhibitory effects of recombinant IL-9 (72–74). Infected cells were treated with IL-9 (10 ng/ml) or control IL-2 (20U/ml) at 37°C with 5% CO2 for 72h to evaluate the inhibitory effects for soluble IL-9.

### 4.10 Adoptive cell transfers and aerosol challenges

For adoptive transfer experiments, naïve CD4+ T cells were isolated from the spleens of ESAT6-specific TCR Tg mice. Cells were differentiated into Th1 and Th9 cells as described above, and then 5x10^6^ cells were injected i.v. into Rag1/2^-/-^ recipients. The following day, groups of mice (N = 8–10 per group) were challenged with virulent Mtb Erdman via aerosol exposure. Mtb was thawed and 6 ml of 2 × 10^6^ CFU/ml loaded into the nebulizer attached to the Glas-Col (Terre Haute, IN) Inhalation Exposure System (IES) based on previously described methods (75). IES settings utilized for the aerosol challenges were: (1) 15-minute preheat, (2) 40-minute nebulization, (3) 40-minute cloud decay, (4) 15-minute UV decontamination, (5) 50 psi vacuum, and (6) 10 psi compressor. Two to five animals per group were euthanized immediately post-aerosol exposure to quantitate the delivery dosage. The above settings resulted in reproducible seeding of ~150 CFU per animal.

### 4.11 Quantitation of viable mycobacteria in tissues

Methods for determining bacterial CFU counts with organ tissue homogenates were described previously (75). Briefly, bacterial CFU were calculated by serial dilution and plating of aliquots of the homogenized organ suspensions. The initial infective dose was verified by sacrificing mice 24 h after aerosol exposure. Tissues were aseptically removed, rinsed in PBS, and placed into 2 mL tubes containing 2-mm solid-glass beads (Sigma) and 1 mL of ADC-supplemented 7H9 Middlebrook media. Samples were homogenized using a Bead Mill 24 (Fisher Scientific) for 3 cycles, 20 seconds each, at 5.5 m/s with 10 second dwell times between cycles. Ten-fold serial dilutions of homogenized tissues were plated on OADC-supplemented 7H10 Middlebrook agar plates -. Plates were incubated at 37°C, and CFU were enumerated after 21 days. To assess protection, mice were euthanized 4 weeks post-infection, and their lungs and spleens were aseptically removed. Half of each organ was homogenized in 1 ml of ADC-supplemented 7H9 Middlebrook media using a Tissue-Tearor (BioSpec) for CFU enumeration as described above. The remaining lung tissues were utilized for other purposes as described below. Data is presented as the meaning of log10 CFU per organ, and SD is indicated by error bars (n=8–10 mice/group).

### 4.12 Histopathology

Murine lungs were prepared by fixing the tissue in 10% buffered formalin before paraffin embedment. Sections were stained with H&E for evaluation of pathological changes. Histopathological analysis of tissues from adoptive transferred and control mice was conducted 4 weeks after aerosol challenge.

### 4.13 BAL from Mice

Bronchoalveolar lavage (BAL) was performed to collect cells for use in flow cytometric analyses as described (76), and BAL fluid prepared for IL-9 ELISA. Briefly, mice were euthanized, and the trachea was exposed and cannulated. The lungs were lavaged three times with 1ml PBS. BAL supernatant was collected for IL-9 ELISA assay. Red blood cells were lysed in NH4Cl lysis buffer, then cells were washed with PBS, before being resuspended in complete media. Then BAL cells were prepared for analysis of cell surface and intracellular markers as described below.

### 4.14 FACS analysis of cell surface markers and intracellular cytokine staining

The cells were stained with the following Abs (Biolegend): CD3 (anti-CD3-Pac blue, clone 500A2), CD4 (anti-CD4-Percp cy5.5, clone RM4-5), CD45 (anti-CD45-PE-cy7, clone 30-F11), TCR-Vβ6 (anti- TCR-Vβ6, clone RR4-7. All staining procedures were performed in PBS containing 0.1% BSA and 0.1% sodium azide (FACS buffer) for 20 min at 4°C. For intracellular cytokine staining, cells were stimulated with PMA (Sigma-Aldrich) and ionomycin (EMD Millipore) for 2.5 h followed by GogiStop (BD Immunocytometry Systems, San Jose, CA) for a total of 4 h. For cytokine staining, cells were fixed with Foxp3/Transcription factor fixation buffer (BD) at 4 °C in dark for 30 min. Fixed cells were permeabilized with permeabilization buffer (BD) and stained for cytokines with fluorochrome-conjugated antibodies (1:200 dilution) at 4 °C in the dark for 1 h. Stained cells were washed two times with FACS buffer and resuspended with 500 μl of FACS buffer for flow analysis. Fluorescent antibodies for cytokine staining are all from Biolegend: including IL-9 (anti IL-9-APC, clone RM9A4), IL-10 (anti-IL-10-FITC, clone JES5-16E3), IFN-γ (anti- IFN-γ-AF700, clone XMG1.2). Cells were fixed with 4% paraformaldehyde for at least 1 h and analyzed by flow cytometry using FlowJo software V10 (Tree Star, Inc., Ashland, OR USA).

### 4.15 cDNA synthesis and qRT-PCR

RNA samples from lung homogenates were extracted using TRIzol reagent (Ambion) and RNeasy spin columns according to manufacturer’s instructions (RNeasy kit, Qiagen). RNA was reverse transcribed into cDNA using M-MLV Reverse transcriptase (Invitrogen). RT-qPCR was performed using gene-targeted primers (**Supplementary Table 1**) as described above. Values were normalized using the GAPDH housekeeping gene and expressed as a fold change in experimental samples (antibiotics-treated mice) relative to control samples (non-treated mice).

### 4.16 Statistical analysis

Graphics and statistical results were generated using GraphPad Prism version.9 (GraphPad Software, La Jolla, CA, USA). Statistical significance was determined using Mann-Whitney U tests or Wilcoxon matched pairs test. The value of p <0.05 was considered significant.

## Data Availability

The datasets presented in this study can be found in online repositories. The names of the repository/repositories and accession number(s) can be found below: https://www.ncbi.nlm.nih.gov/geo/, GSE224055 (Blood) https://www.ncbi.nlm.nih.gov/geo/, GSE223999 (BAL).
