# Supplementary material for: Th9 cells provide protective TB immunity
Source: Front Immunol. 2025 Oct 6;16:1581286. doi: 10.3389/fimmu.2025.1581286 (PMC12535965; doi:10.3389/fimmu.2025.1581286)
Supplement: Supplementary file 1 [file DataSheet1.docx]

Supplementary Material

# Supplementary Tables

Table S1 List of Primers Used in This Study, Related to the Experimental Procedures.

**Primers Used for Quantitative Real-Time PCR**

| Gene | Forward Primer | Reverse Primer |
| --- | --- | --- |
| **Mouse IL-9** | 5’CATCAGTGTCTCTCCGTCCCAACTGATG3’ | 5’-GATTTCTGTGTGG CATTGGTCAG-3’ |
| **Mouse IL-10** | 5’CCCCGCCTGTACTGTAGGAA 3’ | 5’TTCCTACAGTACAGGCGGGG3’ |
| **Mouse PU.1** | 5’ AGTCATCCGATGGAGGGG3’ | 5’ TGGAGCTCAGCTGGATGTTAC3’ |
| **Mouse IFN-γ** | 5’ATGAACGCTACACACTGCATC3’ | 5’CCATCCTTTTGCCAGTTCCTC3’ |
| **Mouse T-bet** | 5’TTTCCAAGAGACCCAGTTCAT3’ | 5’ATGCGTACATGGACTCAAAGT3’ |
| **Mouse IRF-4** | 5’TGAAAATGGTTGCCAGGTGACAGG3’ | 5’ GCAGCCTTCAGGGCTCGTCG3’ |
| **Mouse CCR3** | 5’TCAACTTGGCAATTTCTGACCT3’ | 5’CAGCATGGACGATAGCCAGG3’ |
| **Mouse CXCR3** | 5’GTGCTAGATGCCTCGGACTT3’ | 5’GAGGCGCTGATCGTAGTTGG3’ |
| **Mouse CCR6** | 5’TCTGCACTAGTGAGAGTGTG3’ | 5’GTCATCACCACCATAATGTTG3’ |

# Supplementary Figures

**Supplementary Figure 1**


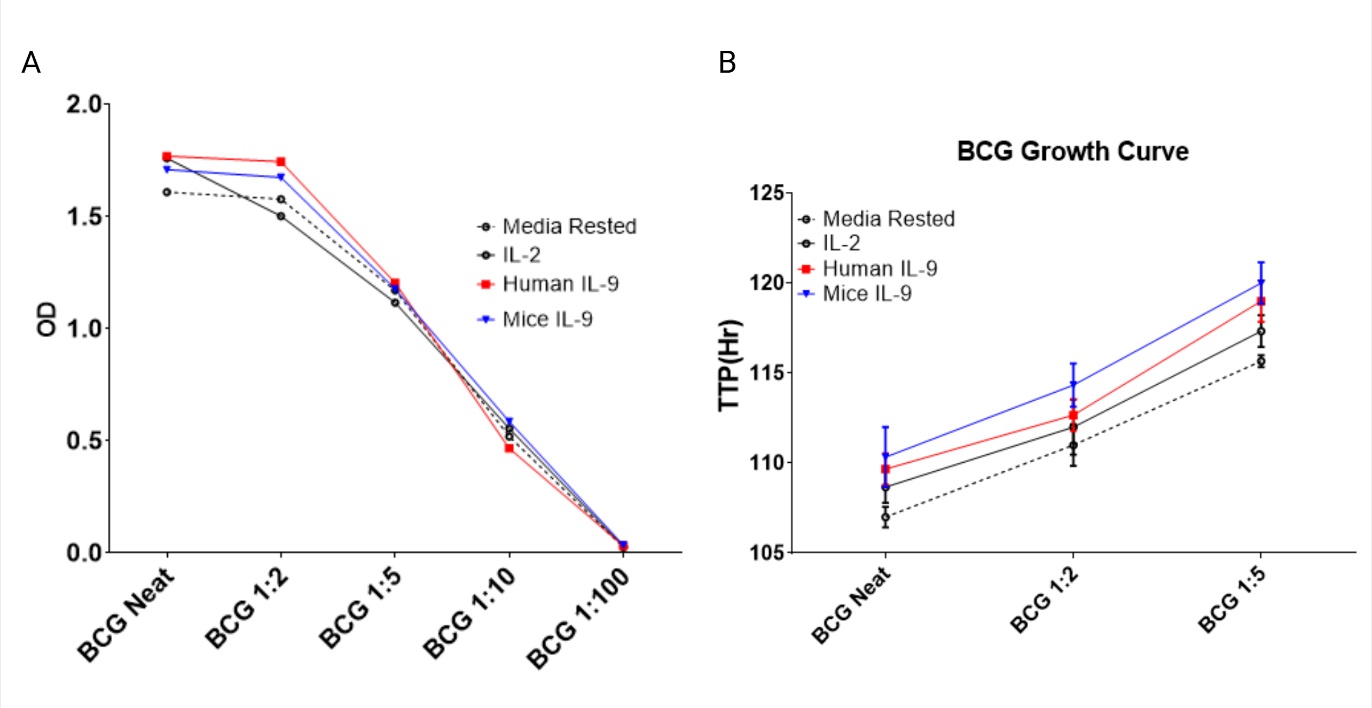


**Supplementary Figure 1.** **IL-9 has no toxic or growth inhibitory effects on extracellular BCG.** In SF1A, the toxicity of IL-9 for BCG was studied using the MTT viability assay. The procedure, adapted from Mshana ([Mshana Robert et al., 1998](#_ENREF_30)) and Abate ([Abate et al., 2004](#_ENREF_1)), involved incubating BCG in 96-well plates at various concentrations in 7H9 OADC medium. Different wells were treated with human recombinant IL-9 (20 μg/ml), murine IL-9 (10 μg/ml) or human IL-2 (20 U/ml) as a control. Each experimental condition was performed in triplicate. MTT (Sigma, St. Louis, Mo.) was prepared in PBS (pH 7.2) to a concentration of 5 mg/ml. Ten microliters of the MTT solution were added to each well, and the plates were incubated at 37°C for 4 hours. Following incubation, 50 microliters of a lysing buffer containing 20% sodium dodecyl sulfate in 50% N-dimethylformamide (pH 4.7) was added. After overnight incubation, absorbance at 570 nm was read using an automatic enzyme-linked immunosorbent assay reader (Flow Laboratories, Irvine, United Kingdom). Shown are mean responses for each condition. In SF1B, the effects of IL-9 on the growth of extracellular BCG alone were studied. Titrations of BCG using two-fold dilutions were inoculated into BD BACTEC^TM^ MGIT^TM^ culture tubes with or without murine/human IL-9 and the time-to-positivity (TTP) measured in a BD BACTEC^TM^ MGIT^TM^ Automated Mycobacterial Detection System. Triplicate means and standard errors are shown. Neither human or murine recombinant IL-9 exhibited any inhibitory effects on BCG growth.

**Supplementary Figure 2**


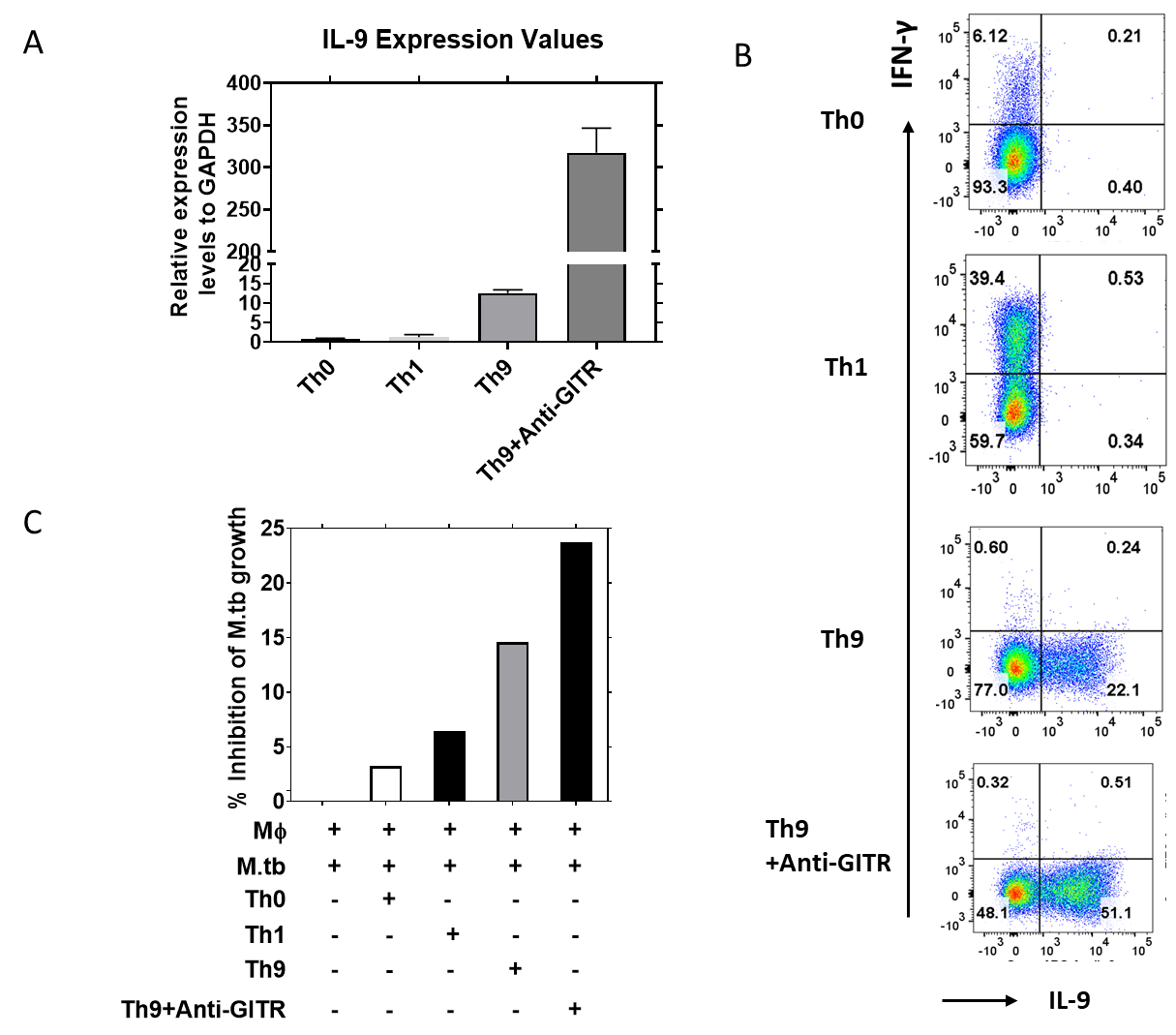


**Supplemental Figure 2. GITR ligation enhances Th9-mediated anti-TB immunity in vitro**. Panel A, qRT-PCR analysis of IL-9 mRNA in ESAT6-specific TCR Tg CD4+ T cells activated for 3 days under Th9-polarizing conditions with agonistic anti-GITR or control IgG. Data shown are representative of two independent experiments. Panel B, purified naïve CD4+ T cells were activated with anti-CD3 and anti-CD28 in the presence of polarizing cytokines specific for Th9 with or without anti-GITR. Intracellular expression of IFN-γ and IL-9 was measured by ICS flow cytometry at day 3. Data from 1 representative experiment. Panel C, naïve CD4+ T cells from ESAT6-specific TCR Tg mice were incubated with Th9- differentiating cytokines (TGFβ1, IL-4 and anti-IFN-γ) with or without anti-GITR stimulation to generate Th9 cells. BMDM cultured from ESAT-6 TCR Tg mice were infected with Mtb at an MOI of 1 overnight. Extracellular bacteria were washed off and freshly differentiated Th9 cells were co-cultured with Mtb*-*infected macrophages at an E:T ratio of 1:1. Mtb viability was determined 3 days later by [^3^H]uridine incorporation. GITR ligation enhanced Th9-mediated anti-TB immunity in vitro.
